# Indobufen Versus Aspirin Plus Clopidogrel in Patients After Coronary Stenting in Patients With Diabetes: A Post Hoc Analysis of the OPTION Trial

**DOI:** 10.1111/1753-0407.70152

**Published:** 2025-09-05

**Authors:** Shujing Wu, Huajie Xu, Lili Xu, Huanyi Zhang, Kang Cheng, Xiaoyan Wang, Manhua Chen, Guangping Li, Jiangnan Huang, Jun Lan, Guanghe Wei, Xin Zhao, Zhiyong Qi, Juying Qian, Hongyi Wu, Junbo Ge, the OPTION investigators

**Affiliations:** ^1^ Department of Cardiology, Zhongshan Hospital Fudan University, Shanghai Institute of Cardiovascular Diseases, National Clinical Research Center for Interventional Medicine Shanghai China; ^2^ Department of Cardiology Zhongshan Hospital (Xiamen), Fudan University Xiamen China; ^3^ Department of Infectious Disease Zhongshan Hospital, Fudan University Shanghai China; ^4^ Department of Cardiology Shanghai Geriatric Medical Center Shanghai China; ^5^ Department of Cardiology Taian City Central Hospital Taian China; ^6^ Department of Cardiology Xi'an no. 3 Hospital, the Affiliated Hospital of Northwest University Xi'an China; ^7^ Department of Cardiology Affiliated Hospital of Jiangnan University Wuxi China; ^8^ Department of Cardiology The Central Hospital of Wuhan Wuhan China; ^9^ Department of Cardiology The Second Hospital of Tianjin Medical University Tianjin China; ^10^ Department of Cardiology The First Affiliated Hospital of Guangxi Medical University Nanning China; ^11^ Department of Cardiology Dongguan Third People's Hospital Dongguan China; ^12^ Department of Cardiology Affiliated Hospital of Jining Medical University Jining China

**Keywords:** aspirin, coronary artery disease, diabetes, dual antiplatelet therapy, indobufen, percutaneous coronary intervention

## Abstract

**Background:**

Despite increased risk of ischemic events in diabetes, the optimal anti‐thrombotic strategy for secondary prevention has not been defined. We aimed to assess the efficacy and safety of optimal antiplatelet agents such as indobufen‐based dual antiplatelet therapy (DAPT) in patients with diabetes after coronary stenting.

**Methods:**

OPTION trial was a randomized, open‐label, noninferiority, and multicentric study in China. Enrolled subjects were randomized 1:1 to indobufen‐based DAPT or aspirin‐based DAPT. This post hoc analysis from OPTION trial was performed by the presence of diabetes. The primary endpoint was a 1‐year composite of cardiovascular death, nonfatal myocardial infarction, ischemic stroke, definite or probable stent thrombosis, or Bleeding Academic Research Consortium (BARC) criteria type 2, 3, or 5 bleeding.

**Results:**

Of 4551 OPTION patients, the primary endpoint occurred in 93/1570 patients with diabetes (5.92%), as compared to 148/2981 without diabetes (4.96%) (HR: 0.72, 95% CI: 0.47–1.08, and HR: 0.73, 95% CI: 0.53–1.01, respectively), without significant interaction between diabetes status and treatment effect (*P*_interaction_ = 0.935). The secondary efficacy endpoint was comparable between patients with (HR: 1.31, 95% CI: 0.60–2.84) and without diabetes (HR: 0.95, 95% CI: 0.51–1.76) (*P*_interaction_ = 0.526). Similarly, both subgroups derived similar benefits for the safety endpoint (HR, 0.56; 95% CI, 0.34–0.92 for subjects with diabetes vs. HR, 0.66; 95% CI, 0.45–0.98 for those without diabetes; *P*_interaction_ = 0.609).

**Conclusions:**

In patients receiving DES implantation, indobufen‐based DAPT might be considered as a reasonable alternative to aspirin‐based DAPT in the secondary prevention for those with diabetes, especially in patients at high bleeding risk.

## Graphical abstract

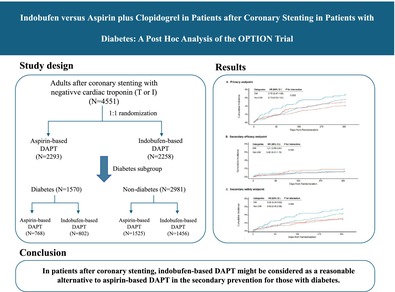

**Summary**

- This post hoc study evaluates risks of both ischemic and bleeding outcomes in patients with diabetes after coronary stenting between a novel indobufen‐based DAPT and traditional aspirin‐based DAPT, filling a critical knowledge gap.
- Results indicate that indobufen‐based DAPT was not different from aspirin‐based DAPT for 1‐year composite endpoints and tended to be associated with decreased bleeding events in patients with diabetes after coronary stenting, regardless of baseline glucose levels and control.
- Indobufen‐based DAPT could be considered a reasonable alternative option to diabetic patients after coronary stenting with aspirin‐based DAPT, especially in patients at high bleeding risk.

## 1 Introduction

Despite advances in therapy, cardiovascular disease (CVD) still remains a main cause of death [1]. Diabetes is a common comorbidity with CVD and correlates with worse clinical outcomes among patients undergoing coronary stenting [2]. Diabetes is considered to be a prothrombotic condition, which develops secondary to platelet hyperreactivity coupled with hypofibrinolysis [3, 4, 5]. Dual antiplatelet therapy (DAPT) is recommended as the cornerstone of treatment in diabetic patients after coronary stent implantation [6]. Of concern is the observation that the cardiovascular prevention of antiplatelet therapy is lower in diabetes than in the general population [7, 8]. Moreover, the impact on the risk of bleeding complications should be considered in the long‐term treatment with DAPT [9]. Thus, the balance between antithrombotic protection and bleeding‐related harm plays a critical role in the DAPT strategies, including in patients with diabetes.

Recently, the OPTION (randomized controlled trial of indObufen versus asPirin after coronary drug‐eluting stent implantaTION) trial showed that in the 12 months follow‐up of patients receiving drug‐eluting stent (DES) implantation, indobufen plus clopidogrel DAPT improved the secondary safety outcome but not at the expense of increasing the secondary efficacy outcome, as compared with aspirin plus clopidogrel DAPT [10]. Therefore, in this post hoc analysis of the OPTION trial, we sought to assess whether patients with diabetes derive similar benefit.

## 2 Methods

### 2.1 Study Design and Population

The design and protocol of the OPTION trial have been reported in detail previously [10]. In brief, the OPTION trial was a randomized, open‐labeled, prospective, noninferiority, and multicentric study in China to investigate the potential aspirin replacement on top of a P2Y12 inhibitor in patients with negative cardiac troponin after DES implantation. Between January 11, 2018 and October 12, 2020, a total of 4551 participants were randomized in a 1:1 ratio to receive indobufen or aspirin‐based DAPT treatment for 12 months.

The trial was approved by the Ethics Committee at each clinical site before initiating the investigation and conducted in accordance with the Declaration of Helsinki. All subjects provided written and informed consent before any trial‐related activities. This study is registered at https://www.chictr.org (registration number: ChiCTR‐IIR‐17013505).

### 2.2 Definitions and Endpoints

Diabetes was defined as a previous diagnosis of diabetes by physicians or a hemoglobin A1c (HbA1c) ≥ 48 mmol/mol (6.5%) without prior known diabetes [11].

The primary endpoint was a composite of cardiovascular (CV) death, nonfatal myocardial infarction (MI), ischemic stroke, definite or probable stent thrombosis (ST), or Bleeding Academic Research Consortium (BARC) criteria type 2, 3, or 5 bleeding during the 12 months follow‐up period. The secondary efficacy endpoint was a composite of CV death, nonfatal MI, ischemic stroke, definite or probable ST. The secondary safety endpoint included BARC criteria type 2, 3, or 5 bleeding. Detailed definitions of each outcome have been described previously [10]. All endpoints were verified by an independent Clinical Event Committee whose members were blinded to the trial group assignments.

### 2.3 Statistical Analysis

Data were presented as means and standard deviations for continuous variables, and numbers and proportions for categorical variables. Baseline characteristics between groups were compared using the unpaired *t* test for continuous variables and the chi‐square test for categorical variables. Analyses were performed on the intention‐to‐treat population (Supporting Information Figure S1).

Time to first event data were compared using the log‐rank test and Kaplan–Meier censoring estimates. For each endpoint, a two‐sample 95% binomial confidence interval (CI) was calculated for the difference in proportions between groups (Difference% = diabetes% minus nondiabetes%, diabetes group and nondiabetes group). Hazard ratios (HRs) with 95% CIs were determined using Cox proportional hazards regression models with interaction testing according to diabetes status. In the patients with diabetes, sensitivity analyses were performed according to baseline HbA1c levels (achieving an optimal target or not, < 7.0% or 7.0%) and random glucose levels (less than or no less than mean levels, < 8.25 mmol/L or 8.25 mmol/L). Interaction terms were also used in the sensitivity analyses to test for baseline HbA1c levels or blood glucose levels by intervention group interactions.

A 2‐sided *p* value < 0.05 was considered statistically significant. All analyses were conducted using Stata (version 18.0; StataCorp) and R (version 4.3.1; The R Foundation for Statistical Computing).

## 3 Results

### 3.1 Baseline Characteristics

In OPTION trail, 1570 out of 4551 patients (34.5%) had diabetes at baseline (802 in the indobufen‐based DAPT group and 768 in the aspirin‐based DAPT group) (Supporting Information Table S1). Compared with subjects without diabetes, those with diabetes had increased proportions of women, hypertension, hyperlipidemia, nonsmokers currently, previous history of CVDs, the academic research consortium for high bleeding risk (ARC‐HBR), multivessel disease, complex percutaneous coronary intervention (PCI), use of β‐blockers, angiotensin‐converting‐enzyme inhibitor (ACEI) or angiotensin II receptor blocker (ARB), higher levels of body mass index (BMI), a greater number of implanted stents, longer lengths of stents, and lower levels of hemoglobin (Table 1). In addition, the two treatment groups were well balanced for most baseline characteristics in both patients with and without diabetes (Supporting Information Table S1). Among participants with diabetes, those receiving indobufen‐based DAPT were more likely to have hyperlipidemia than those receiving aspirin‐based DAPT. Among participants without diabetes, the indobufen group had increased proportions of men and previous history of stroke, and had a lower proportion of taking proton pump inhibitor (PPI), as compared with the aspirin group (Supporting Information Table S1).

**TABLE 1:** Baseline characteristics by diabetes status.

	Diabetes (*n* = 1570)	Nondiabetes (*n* = 2981)	*p*
Age, y	61.3 ± 8.1	61.0 ± 8.4	0.330
Male	967 (61.6)	2001 (67.1)	< 0.001
BMI, kg/m^2^	25.5 ± 3.1	24.8 ± 3.3	< 0.001
Hypertension	1193 (76.0)	1866 (62.6)	< 0.001
Hyperlipidemia	601 (38.3)	877 (29.4)	< 0.001
Current smoking	360 (22.9)	765 (25.7)	0.046
Previous myocardial infarction	123 (7.8)	151 (5.1)	< 0.001
Previous heart failure	120 (7.6)	150 (5.0)	< 0.001
Previous stroke	105 (6.7)	149 (5.0)	0.022
Previous gastrointestinal bleeding	12 (0.8)	16 (0.5)	0.463
ARC‐HBR	120 (7.6)	162 (5.4)	0.004
Unstable angina	889 (56.6)	1679 (56.3)	0.870
Hemoglobin, g/L	137.6 ± 15.7	139.5 ± 14.9	< 0.001
Blood glucose, mmol/L	8.2 ± 3.7	5.4 ± 1.1	< 0.001
HbA1c, %	7.8 ± 4.3	5.7 ± 0.4	< 0.001
LDL‐c, mmol/L	2.5 ± 1.0	2.6 ± 0.9	0.124
Serum creatinine, μmol/L	72.1 ± 22.1	73.2 ± 17.9	0.099
Creatinine clearance < 60 mL/min	201 (12.8)	379 (12.7)	0.969
Multivessel disease	970 (61.8)	1574 (52.8)	< 0.001
No. of stents	1.6 ± 0.8	1.5 ± 0.8	0.001
Length of stents, mm	39.8 ± 23.8	37.3 ± 24.0	< 0.001
Bifurcation target lesion	147 (9.4)	278 (9.3)	> 0.999
Complex PCI	429 (27.3)	695 (23.3)	0.003
Statin	1468 (93.5)	2753 (92.4)	0.173
β‐Blocker	1137 (72.4)	2009 (67.4)	< 0.001
ACE inhibitor or ARB	996 (63.4)	1654 (55.5)	< 0.001
PPIs	762 (48.5)	1461 (49.0)	0.784

*Note:* Data are expressed as mean ± SD or number of patients (percentage).

Abbreviations: ACE, angiotensin‐converting‐enzyme inhibitor; ARB, angiotensin II receptor blocker; ARC‐HBR, academic research consortium for high bleeding risk; BMI, body mass index; LDL‐c, Low‐density lipoprotein cholesterol; PCI, percutaneous coronary intervention; PPI, proton pump inhibitor.

### 3.2 Outcomes Based on Diabetes Status

During 12 months follow‐up, the event rates of the primary endpoint in patients with and without diabetes were 5.92% (93 of 1570) and 4.96% (148 of 2981), respectively (Table 2). The absolute difference for the primary endpoint with diabetes vs. nondiabetes was 0.96% (−0.45% to 2.36%) and the rates were not significantly different between diabetes and nondiabetes (HR, 1.20; 95% CI, 0.93–1.56; *p* = 0.162) (Table 2). Similarly, no statistically significant differences were found between diabetes and nondiabetes in the incidence of the secondary efficacy endpoint, secondary safety endpoint, and individual endpoints (Table 2).

**TABLE 2:** 1‐year clinical endpoint according to presence of diabetes.

	Diabetes (*n* = 1570)	Nondiabetes (*n* = 2981)	Difference% (95% CI)	HR (95% CI)	*p*
Primary endpoint^a^	93 (5.92)	148 (4.96)	0.96 (−0.45 to 2.36)	1.20 (0.93, 1.56)	0.162
Secondary efficacy endpoint^b^	26 (1.66)	40 (1.34)	0.31 (−0.44 to 1.07)	1.24 (0.76, 2.03)	0.397
Cardiovascular death	4 (0.25)	3 (0.10)	0.15 (−0.12 to 0.43)	2.54 (0.57, 11.34)	0.223
Nonfatal MI	7 (0.45)	12 (0.40)	0.04 (−0.36 to 0.44)	1.11 (0.44, 2.82)	0.826
Ischemic stroke	13 (0.83)	24 (0.81)	0.02 (−0.53 to 0.57)	1.03 (0.53, 2.03)	0.927
Definite or probable stent thrombosis	5 (0.32)	4 (0.13)	0.18 (−0.12 to 0.49)	2.38 (0.64, 8.86)	0.196
Secondary safety endpoint^c^	67 (4.27)	108 (3.62)	0.64 (−0.56 to 1.85)	1.19 (0.87, 1.61)	0.276
BARC type 3, 5 bleeding	25 (1.59)	32 (1.07)	0.52 (−0.20 to 1.24)	1.49 (0.88, 2.52)	0.134
BARC type 2 bleeding	42 (2.68)	76 (2.55)	0.13 (−0.85 to 1.10)	1.06 (0.72, 1.54)	0.779
BARC type 3 bleeding	23 (1.46)	25 (0.84)	0.63 (−0.05 to 1.30)	1.76 (1.00, 3.10)	0.051
BARC type 5 bleeding	2 (0.13)	7 (0.23)	−0.11 (−0.36 to 0.14)	0.54 (0.11, 2.62)	0.448

*Note:* Data are expressed as number of patients (percentage).

Abbreviations: BARC, Bleeding Academic Research Consortium; CI, confidence interval; HR, hazard ratio; MI, myocardial infarction.

^a^ The primary endpoint was a composite of cardiovascular death, nonfatal MI, ischemic stroke, definite or probable stent thrombosis, or BARC criteria type 2, 3, or 5 bleeding.

^b^ The secondary efficacy endpoint was a composite of cardiovascular death, nonfatal MI, ischemic stroke, definite or probable stent thrombosis.

^c^ The secondary safety endpoint included BARC criteria type 2, 3, or 5 bleeding.

### 3.3 Outcomes Based on Diabetes Status and Randomized Intervention Assignment

**FIGURE 1:**
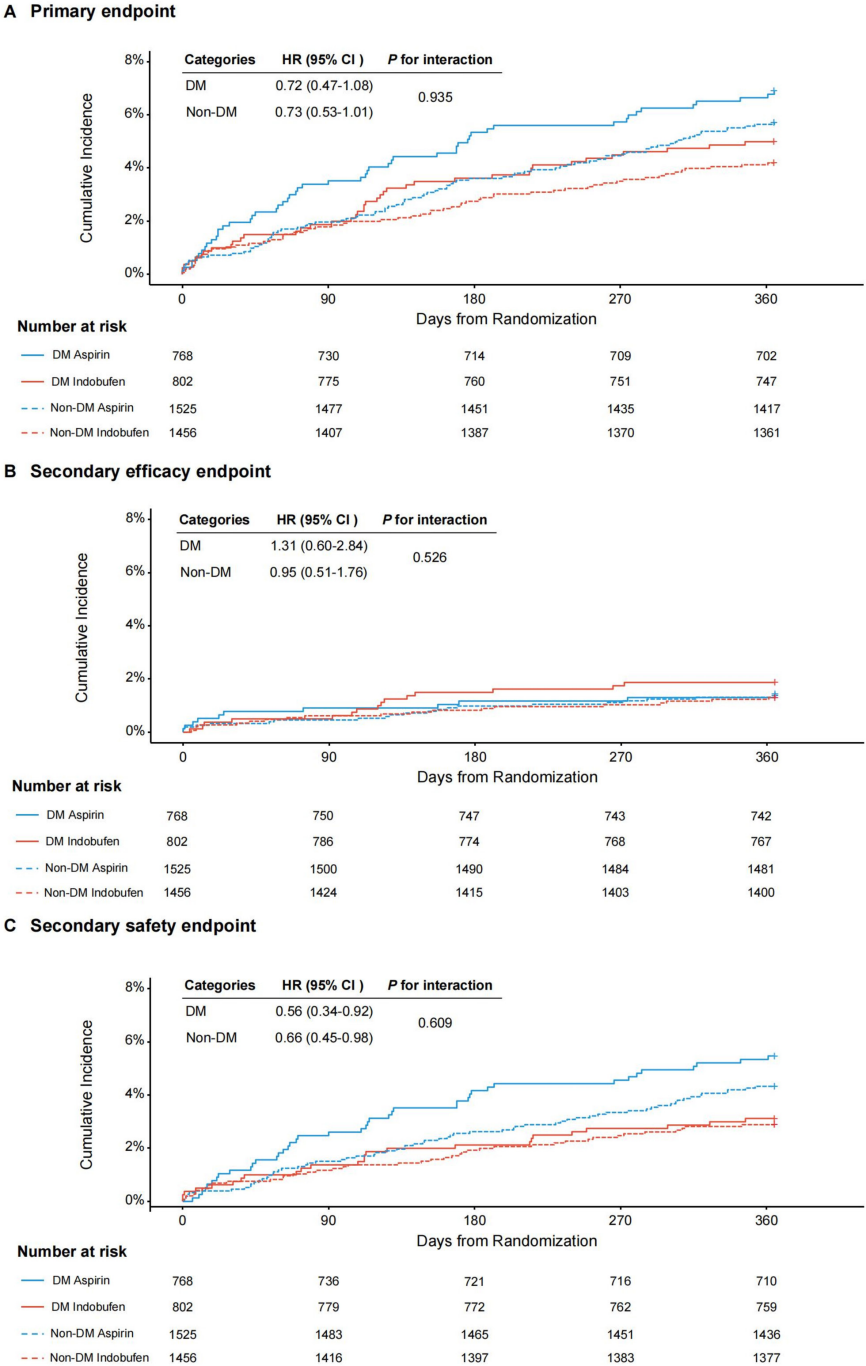
Kaplan–Meier curves of the clinical endpoints in the intention‐to‐treat population according to diabetes status. (A) Primary endpoint (a composite of cardiovascular death, nonfatal MI, ischemic stroke, definite or probable stent thrombosis, or BARC criteria type 2, 3 or 5 bleeding). (B) Secondary efficacy endpoint (a composite of cardiovascular death, nonfatal MI, ischemic stroke, definite or probable stent thrombosis). (C) Secondary safety endpoint (BARC criteria type 2, 3 or 5 bleeding events). HR, hazard ratio; CI, confidence interval; MI, myocardial infarction; BARC, Bleeding Academic Research Consortium.

The primary endpoint for indobufen‐based DAPT versus aspirin‐based DAPT in those with and without diabetes was shown in Figure 1 and Supporting Information Table S2. The risks of the primary endpoint did not differ by the presence of diabetes without significant interaction (*p* for interaction = 0.935). Compared with the aspirin group, the indobufen group was associated with a numerically lower but not statistically significant risk of the primary endpoint in diabetes (HR, 0.72; 95% CI, 0.47–1.08) and those without diabetes (HR, 0.73; 95% CI, 0.53–1.01) (Figure 1A and Supporting Information Table S2). In terms of the secondary efficacy endpoint, the HR (CIs) with the indobufen group was 1.31 (0.60–2.84) in patients with diabetes and 0.95 (0.51–1.76) in patients without diabetes (*p* for interaction = 0.526) (Figure 1B and Supporting Information Table S2). Although the magnitude of reduction with the indobufen group in risks for the secondary safety endpoint appeared larger in subjects with vs. without diabetes, both subgroups derived similar benefit (HR, 0.56; 95% CI, 0.34–0.92 for subjects with diabetes vs. HR, 0.66; 95% CI, 0.45–0.98 for those without diabetes; *p* for interaction = 0.609), regardless of diabetes status (Figure 1C and Supporting Information Table S2).

When endpoints were examined separately, we also observed similar patterns in participants with and without diabetes (Supporting Information Table S2). No significant association was noted between randomized intervention assignment and diabetes status for individual endpoints (all *p* for interaction > 0.10) (Supporting Information Table S2). We noted that the rates of BARC 2 bleeding were significantly lower in the indobufen group than in the aspirin group in participants with and without diabetes (Supporting Information Table S2).

**FIGURE 2:**
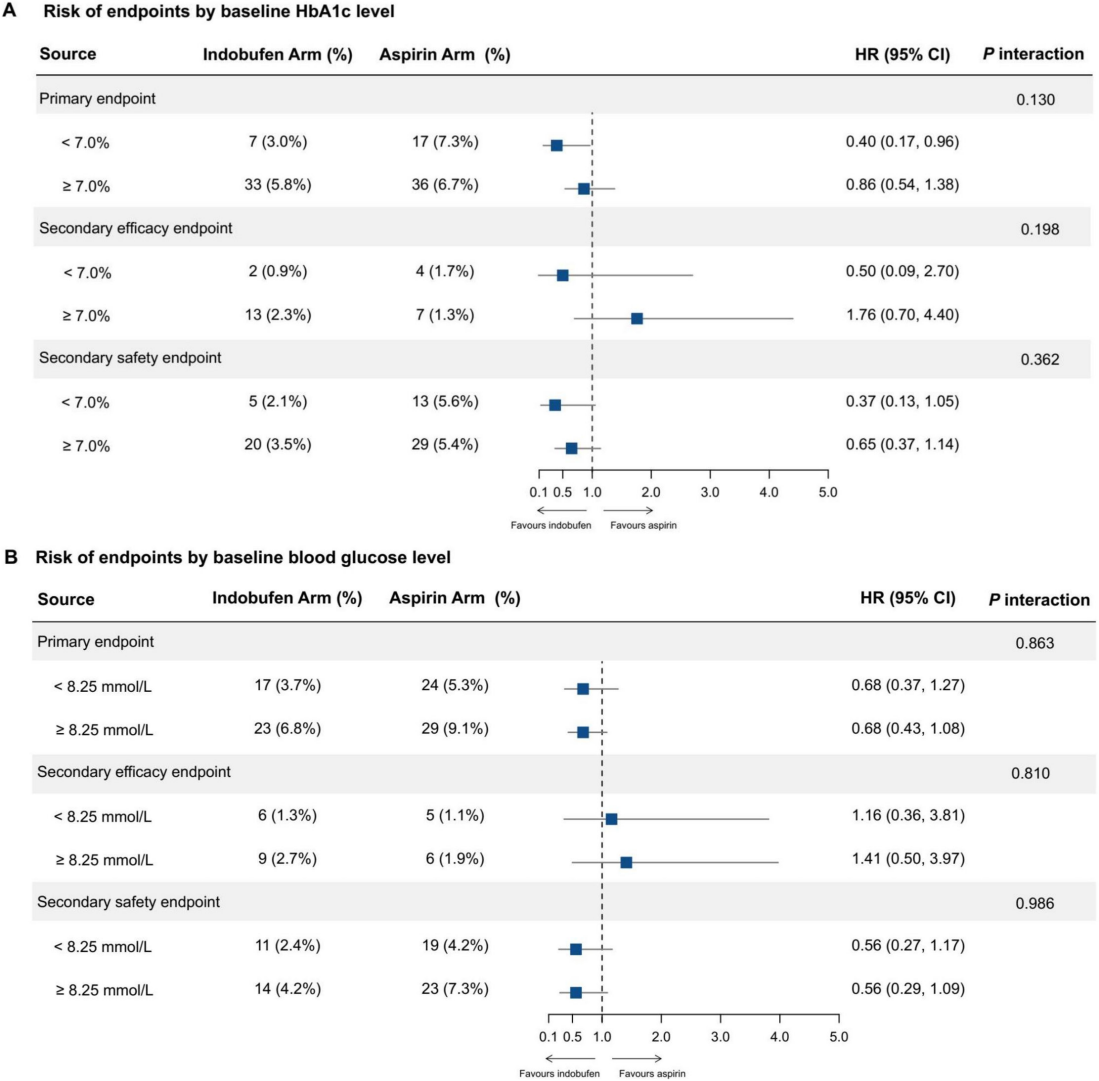
Risk of primary and secondary endpoints according to subsets related with baseline HbA1c (A) and blood glucose (B) levels in participants with diabetes. HR, hazard ratio; CI, confidence interval; HbA1c, hemoglobin A1c.

Likewise, sensitivity analyses using different glycemic levels in participants with diabetes yielded consistent results for the primary and secondary outcomes (Figure 2). When participants with diabetes were subdivided according to baseline HbA1c and random glucose levels, the indobufen group was associated with lower rates of the primary endpoint in those achieving an optimal HbA1c target of less than 7% at baseline (Figure 2A). However, the effect of the indobufen group compared with the aspirin group was consistently observed across all glycemic levels with no significant interaction (Figure 2).

## 4 Discussion

In this post hoc analysis from the OPTION trial, indobufen plus clopidogrel DAPT for 12 months tended to decrease the risk of the primary endpoint in patients with diabetes after coronary stenting. Additionally, indobufen‐based DAPT suggested a potential benefit in terms of 12‐month bleeding events without a concomitant increase in ischemic events among participants with diabetes, which might be mainly driven by reducing the risk of BARC type 2 bleeding.

Diabetes has been widely recognized as a prothrombotic state [3, 4, 5]. Patients with diabetes after PCI had an increased risk of long‐term mortality and cardiovascular events [12, 13]. In addition, post‐PCI bleeding remains an important clinical conundrum of contemporary PCI [14]. Whether diabetes has an increased risk of bleeding complications is still under debate [15, 16, 17, 18]. A multicenter registry study enrolling 4184 subjects and a population‐based cohort study including 4.1 million citizens showed that patients with coronary artery disease who also had diabetes were at higher risk of BARC type ≥ 3 bleeding than the nondiabetic counterparts [15, 16]. The underlying mechanisms remain to be determined and would be as follows: (1) compared with subjects without diabetes, those with diabetes had higher plasminogen activator inhibitor‐1 (PAI‐1) and tissue‐type plasminogen activator (t‐PA) levels, which indicated a disorder in the fibrinolytic system [19]; (2) the glucose‐lowering agents, such as metformin, may interact with antiplatelet drugs to reduce platelet activity [20]. Nevertheless, the current analyses showed inconsistent results. Several studies also failed to confirm the roles of diabetes in elevating the risk of bleeding events among patients undergoing PCI [17, 18]. Given the heterogeneity of diabetes, selecting the optimal antiplatelet therapy with a good balance between thrombosis and bleeding is critical for secondary prevention in these patients.

Of note, diabetes may be associated with reduced clinical efficacy in the platelet inhibitory effects of aspirin [21, 22]. Platelets from patients with diabetes showed increased reactivity, accelerated turnover, and reduced its aspirin‐binding capacity [23, 24]. To the best of our knowledge, there are limited randomized data regarding the optimal antiplatelet regimen with focus on patients with diabetes after PCI. A post hoc analysis including 1860 participants with diabetes found that clopidogrel monotherapy was associated with a lower rate of the primary outcome compared to aspirin monotherapy in patients who have undergone coronary stenting and successfully completed DAPT [25]. Moreover, an emerging paradigm shift favors short‐term DAPT over long‐term DAPT in patients with diabetes undergoing PCI [26, 27]. The search for a more ideal DAPT regimen yet continued. In this case, indobufen, a reversible inhibitor of platelet cyclooxygenase (COX)‐1 activity [28], may be used in those intolerant or allergic to aspirin or in special populations such as those with high risk of bleeding. Our previous study provided head‐to‐head comparison between indobufen‐based and aspirin‐based DAPT [10]. Indobufen group demonstrated equivalent efficacy and better safety relative to aspirin group in patients after coronary DES implantation [10]. In contrast to the irreversible effects of aspirin lasted approximately 1 week [29], the antiplatelet effects of indobufen are short‐lived, and the inhibition of platelet aggregation was almost diminished within 24 h [30, 31]. Thus, indobufen may be more appropriate and effective than aspirin in reducing bleeding risk in patients with diabetes.

Given that HbA1c and glucose levels were considered a reflection of glycemic control and status, they might play a key role in maintaining the balance between thrombosis and bleeding. Elevated levels of HbA1c have been shown to enhance platelet activation in aspirin‐treated patients with coronary artery disease [32, 33]. A previous study conducted on patients with stable coronary diseases showed that, compared with the lower baseline HbA1c levels, the higher baseline HbA1c levels were associated with an increase in the incidence of ischemic events, though higher baseline HbA1c levels were not associated with a higher risk of bleeding events [15]. Several studies assessed patients with DES implantation and reported that patients with higher levels of HbA1c or blood glucose had an elevated risk of ischemic and bleeding outcomes [18, 34]. Still, high‐quality evidence for the association of diabetes status and glycemic control with ischemic and bleeding events in patients receiving different DAPT is lacking. In the present study, we performed sensitivity analyses according to HbA1c and random glucose levels and found that the risks of composite and individual endpoints were comparable between groups, despite limitations of the statistical power.

## 5 Limitations

Several limitations of this study should be considered. First, as subjects in the OPTION trial were not randomized by diabetes status, the statistical power in the current study was insufficient to confirm the difference observed between the two DAPT groups in those with and without diabetes. A lower‐than‐anticipated event rate also resulted in a corresponding loss of statistical power [10]. Second, since the levels of random blood glucose and HbA1c were tested only once at baseline, the newly diagnosed diabetes was defined as HbA1c ≥ 6.5% to reduce misclassification bias. Third, although we found that other covariates were generally balanced between treatment arms, we cannot rule out the possibility that the lack of information on the use of antidiabetic medication and glycemic control during follow‐up may result in limited power to detect associations, since such diabetes‐related covariates (e.g., the use of sodium‐glucose co‐transporter 2 inhibitors) may have interactions with platelet reactivity and cardiovascular outcomes [35]. Fourth, this trial was designed in 2017, before 6‐month DAPT was recommended in patients with chronic coronary syndrome in the Chinese consensus [36]. Therefore, the current results from patients receiving a 12‐month DAPT regimen cannot be extrapolated to those receiving a shorter duration of DAPT. Finally, this trial was conducted solely in the Chinese population, which limits the ethnic generalizability of our findings. Future investigations in broader populations such as Western cohorts are required to reproduce and externally validate the findings in this study.

## 6 Conclusions

In this post hoc analysis of the OPTION trial, during 12‐month DAPT in Chinese patients with negative cardiac troponin after DES implantation, indobufen plus clopidogrel DAPT tended to be associated with a numerically lower but not statistically significant risk of the primary endpoint and might reduce the risk of the secondary safety endpoint without an increased risk of the efficacy endpoint compared to aspirin plus clopidogrel DAPT in patients with diabetes.

## Author Contributions

J.G., J.Q., and H.W. conceived and designed the study. S.W. analyzed the data. H.X. verified the data. S.W., H.X., and L.X. drafted the manuscript. H.W., J.Y.Q., and J.G. revised the manuscript. H.Z., K.C., X.W., M.C., G.L., J.H., J.L., G.W., X.Z., and Z.Q. collected the data and critically revised the manuscript for important intellectual content. All authors read and approved the final manuscript.

## Ethics Statement

The design and primary results of the OPTION trial are published [10]. The trial was conducted in accordance with the Declaration of Helsinki, and local and national regulations. The protocol was approved by the Ethics Committee at each clinical site before initiating the investigation, and all patients provided their written informed consent to participate. This study is registered at https://www.chictr.org (registration number: ChiCTR‐IIR‐17013505).

## Conflicts of Interest

The authors declare no conflicts of interest.

## Supporting information


**Data S1:** jdb70152‐sup‐0001‐Supinfo.docx.

## Data Availability

The datasets used and/or analyzed during the current study are available from the corresponding author on reasonable request.
