# Supplementary material for: Indobufen Versus Aspirin Plus Clopidogrel in Patients After Coronary Stenting in Patients With Diabetes: A Post Hoc Analysis of the OPTION Trial
Source: J Diabetes. 2025 Sep 5;17(9):e70152. doi: 10.1111/1753-0407.70152 (PMC12411781; doi:10.1111/1753-0407.70152)
Supplement: Supplementary file 1 — Data S1: jdb70152‐sup‐0001‐Supinfo.docx. [file JDB-17-e70152-s001.docx]

**Indobufen versus Aspirin plus Clopidogrel in Patients after Coronary Stenting According to Glycemic Status: A Post Hoc Subgroup Analysis of the OPTION Trial**

**Supplementary appendix**

**Contents**

List of investigators for the OPTION (randomized controlled trial of indObufen versus asPirin after coronary drug-eluting stent implantaTION) trial group....................................... 1

**Supplementary Table S1**. Baseline characteristics of the intention-to-treat population by diabetes status............................................................................................................................ 5

**Supplementary Table S2**. 1-year clinical endpoint in the intention-to-treat population according to presence of diabetes............................................................................................. 7

**Supplementary Figure S1**. Study Flowchart.......................................................................... 8

**Investigators for the OPTION (randomized controlled trial of indObufen versus asPirin after coronary drug-eluting stent implantaTION) trial group**

Steering Committee: Junbo Ge MD., Fusui Ji MD., Jianan Wang MD., Zaixin Yu MD., Haichu Yu MM. and investigators from the participating hospitals.

Executive Committee: Junbo Ge MD., Juying Qian MD., Hongyi Wu MD., Lili Xu MD., Xin Zhao MD.

**Investigators in the OPTION trial**

Zhongshan Hospital Fudan University Junbo Ge

Shanghai First People's Hospital Baoshan Branch Minlei Liao

Shanghai Jiao Tong University Affiliated Sixth People’s Hospital Chengxing Shen

Shanghai Ninth People's Hospital, Shanghai Jiaotong University School of Medicine Changqian Wang

Sir Run Run Shaw Hospital, Zhejiang University School of Medicine Guosheng Fu

The Second Affiliated Hospital of Zhejiang University School of Medicine Jian’an Wang

The First Affiliated Hospital of Sun Yat-sen University Xugang Dong

Nanfang Hospital Southern Medical University Yuqing Hou

The Second Affiliated Hospital of Guangzhou Medical University Shiming Liu

Sun Yat-Sen Memorial Hospital, Sun Yat-Sen University Shuxian Zhou

Wuhan Asia Heart Hospital Xi Su

Xiangya Hospital of Central South University Zaixin Yu

Shanxi Cardiovascular Hospital Jian An

Taiyuan Central Hospital of Shanxi Medical University Dengfeng Ma

Shengjing Hospital of China Medical University Xiaodong Li

The Second Hospital of Dalian Medical University Peng Qu

The Affiliated Hospital of Inner Mongolia Medical University Yuexi Wang

Beijing Union Medical College Hospital Hongzhi Xie

Beijing Chaoyang Hospital, Capital Medical University Xinchun Yang

Tengzhou Central People's Hospital Yong Li

The Affiliated Hospital of Qingdao University Haichu Yu

Linyi City People's Hospital Yanjin Wei

Harbin The First Hospital Lin Wei

The Fourth Affiliated Hospital of Harbin Medical University Xueqi Li

Shandong Provincial Hospital Haitao Yuan

Hebei General Hospital Xiaoyong Qi

Jiangsu Province Hospital Xiangqing Kong

Zhongda Hospital Southeast University Genshan Ma

Fujian Provincial Hospital Zhiyong Wu

Xiamen Cardiovascular Hospital Xiamen University Yan Wang

Anhui Provincial Hospital Ji Yan

Tianjin First Central Hospital Xiaofei Wang

First Affiliated Hospital,School of Medicine, Shihezi University Yin Yin

Fujian Medical University Union Hospital Lianglong Chen

Hainan General Hospital Jianlin Ma

Tongren Hospital Shanghai Jiaotong University School of Medicine Chaohui Qiu

Inner Mongolia Autonomous Region People's Hospital Xingsheng Zhao

The People's Hospital of Guangxi Zhuang Autonomous Region Ling liu

The Second Affiliated Hospital of Xi'an Jiaotong University Qiangsun Zheng

China-Japan Friendship Hospital Jingang Zheng

The Third Xiangya Hospital of Central South University Yu Cao

Shaanxi Provincial People's Hospital Junkui Wang

West China Hospital of Sichuan University Lingyun Jiang

Affiliated Hospital of Zunyi Medical University Bei Shi

The Second Xiangya Hospital of Central South University Shenghua Zhou

The Affiliated Hospital of Guizhou Medical University Wei Li

Tongji Hospital, Tongji Medical College, Huazhong University of Science and Technology Hesong Zeng

The First People's Hospital of Yunnan Province Hong Zhang

The Second Affiliated Hospital of Hainan Medical College Xianxia Liu

Zhongnan Hospital of Wuhan University Yanggan Wang

The Central Hospital of Wuhan Manhua Chen

Haikou People's Hospital Shijuan Lu

The First Affiliated Hospital of Chongqing Medical University Suxin Luo

Sichuan Provincial People’s Hospital Jianhong Tao

Tai’an City Central Hospital Huanyi Zhang

Wuhan No.1 Hospital Liqun He

Mianyang Central Hospital Caidong Luo

Beijing Hospital Fusui Ji

Affiliated Hangzhou First People’s Hospital, Zhejiang University School of Medicine Yizhou Xu

The Affiliated Hospital of Northwest University, Xi’an No.3 Hospital Kang Cheng

Guizhou Provincial People’s Hospital Yongyao Yang

Shanghai Pudong New District Zhoupu Hospital Zhongping Ning

The First Affiliated Hospital of Xinxiang Medical College Zhigang Chen

The First Hospital of Hebei Medical University Mingqi Zheng

The Second Affiliated Hospital of Kunming Medical University Wenwei Bai

The Jiangxi Province People’s Hospital Lang Hong

Affiliated Hospital of Jining Medical University Guanghe Wei

The First Affiliated Hospital of Nanchang University Zeqi Zheng

Zhejiang Provincial Hospital of Chinese Medicine Wei Mao

Affiliated Hospital of Jiangnan University Xiaoyan Wang

Huazhong University of Science and Technology Union Shenzhen Hospital Peiyi Xie

Northern Jiangsu People’s Hospital Shenghu He

The Second Affiliated Hospital of Shandong University of Traditional Chinese Medicine Ying Wang

Yiyang Central Hospital Xianming Wu

The Second Hospital of Tianjin Medical University Guangping Li

The Affiliated Hospital of Xuzhou Medical University Zhirong Wang

Dongying People's Hospital Zhenhua Li

Taizhou Hospital of Zhejiang Province Jianjun Jiang

Yancheng No.1 People's Hospital Yunfeng Ju

Xinjiang Production and Construction Corps Hospital Wei Xie

The People's Hospital of Liaoning Province Aijie Hou

Shenzhen University General Hospital Haiying Li

The First Affiliated Hospital of Chengdu Medical College Peijian Wang

Traditional Chinese Medical Hospital of Xinjiang Uygur Autonomous Region Peng Li

Central Hospital Affiliated to Shandong First Medical University Guohai Su

Tianjin Rehabilitation and Recuperation Center Shenghua Ding

Zibo Central Hospital Jun Wang

Peking University Hospital Third Hospital Yanqing Hospital Lixin Wang

The First Affiliated Hospital of Hebei North University Fangjiang Li

Tangshan Gongren Hospital Feng Lu

Binzhou Medical University Hospital Huipu Xu

Linyi Central Hospital Cunyu Fu

Qingdao Municipal Hospital Yibing Shao

The Third Medical Center of Chinese PLA General Hospital Dongxing Ma

Fifth Hospital in Wuhan Lifeng Hong

Yuebei People’s Hospital Liangqiu Tang

Lishui Municipal Central Hospital Chunlai Zeng

The Second Affiliated Hospital of Shandong First Medical University Fenglin Jin

The Third Affiliated Hospital of Qiqihar Medical College Haifeng Shao

The Jilin Heart Hospital Shiqi Liu

The First Bethune Hospital of Jilin University Qian Tong

Dongguan Third People’s Hospital Jun Lan

The First Affiliated Hospital of Guangxi Medical University Jiangnan Huang

**Supplementary Table S1. Baseline characteristics of the intention-to-treat population by diabetes status**

|  | **Diabetes** | | ***P* value** | **Non-diabetes** | | ***P* value** |
| --- | --- | --- | --- | --- | --- | --- |
|  | **Indobufen (n=802)** | **Aspirin (n=768)** |  | **Indobufen (n=1456)** | **Aspirin (n=1525)** |  |
| Age, y | 61.2 ± 8.2 | 61.4 ± 8.1 | 0.633 | 60.9 ± 8.3 | 61.1 ± 8.6 | 0.489 |
| Male | 512 (63.8) | 455 (59.2) | 0.069 | 1009 (69.3) | 992 (65.0) | 0.015 |
| BMI, kg/m^2^ | 25.4 ± 3.0 | 25.6 ± 3.2 | 0.192 | 24.8 ± 3.4 | 24.8 ± 3.2 | 0.687 |
| Hypertension | 606 (75.6) | 587 (76.4) | 0.730 | 911 (62.6) | 955 (62.6) | > 0.999 |
| Hyperlipidemia | 328 (40.9) | 273 (35.5) | 0.033 | 416 (28.6) | 461 (30.2) | 0.341 |
| Current smoking | 608 (75.8) | 602 (78.4) | 0.249 | 1066 (73.2) | 1150 (75.4) | 0.184 |
| Previous myocardial infarction | 737 (91.9) | 710 (92.4) | 0.754 | 1384 (95.1) | 1446 (94.8) | 0.834 |
| Previous heart failure | 66 (8.2) | 54 (7.0) | 0.425 | 62 (4.3) | 88 (5.8) | 0.071 |
| Previous stroke | 60 (7.5) | 45 (5.9) | 0.236 | 86 (5.9) | 63 (4.1) | 0.032 |
| Previous gastrointestinal bleeding | 6 (0.7) | 6 (0.8) | > 0.999 | 7 (0.5) | 9 (0.6) | 0.875 |
| ARC-HBR | 64 (8.0) | 56 (7.3) | 0.676 | 81 (5.6) | 81 (5.3) | 0.824 |
| Unstable angina | 455 (56.7) | 434 (56.5) | 0.970 | 810 (55.6) | 869 (57) | 0.480 |
| Hemoglobin, g/L | 138.3 ± 16.2 | 136.9 ± 15.2 | 0.073 | 139.7 ± 14.8 | 139.3 ± 15 | 0.436 |
| Blood glucose, mmol/L | 8.2 ± 3.6 | 8.3 ± 3.8 | 0.664 | 5.4 ± 1.0 | 5.4 ± 1.1 | 0.921 |
| HbA1c, % | 7.7 ± 1.7 | 7.9 ± 5.9 | 0.427 | 5.7 ± 0.4 | 5.7 ± 0.4 | 0.759 |
| LDL-c, mmol/L | 2.5 ± 1.0 | 2.5 ± 1.0 | 0.630 | 2.6 ± 0.9 | 2.6 ± 1.0 | 0.630 |
| Serum creatinine, μmol/L | 71.8 ± 20.5 | 72.4 ± 23.6 | 0.640 | 73.6 ± 18.2 | 72.8 ± 17.6 | 0.269 |
| Creatinine clearance < 60mL/min | 105 (13.1) | 96 (12.5) | 0.783 | 189 (13.0) | 190 (12.5) | 0.710 |
| Multivessel disease | 506 (63.1) | 464 (60.4) | 0.299 | 758 (52.1) | 816 (53.5) | 0.450 |
| No. of stents | 1.56 ± 0.80 | 1.58 ± 0.83 | 0.657 | 1.48 ± 0.75 | 1.50 ± 0.77 | 0.321 |
| Length of stents, mm | 39.9 ± 23.8 | 39.6 ± 23.9 | 0.749 | 36.6 ± 22.7 | 37.9 ± 25.1 | 0.119 |
| Bifurcation target lesion | 77 (9.6) | 70 (9.1) | 0.807 | 138 (9.5) | 140 (9.2) | 0.829 |
| Complex PCI | 214 (26.7) | 215 (28.0) | 0.599 | 327 (22.5) | 368 (24.1) | 0.300 |
| Statin | 754 (94.0) | 714 (93.0) | 0.460 | 1333 (91.6) | 1420 (93.1) | 0.125 |
| β-Blocker | 580 (72.3) | 557 (72.5) | 0.972 | 957 (65.7) | 1052 (69.0) | 0.063 |
| ACE inhibitor or ARB | 515 (64.2) | 481 (62.6) | 0.549 | 788 (54.1) | 866 (56.8) | 0.154 |
| PPIs | 401 (50.0) | 361 (47.0) | 0.256 | 684 (47.0) | 777 (51.0) | 0.033 |

Data are expressed as mean±SD or number of patients (percentage).
ACE, angiotensin-converting-enzyme inhibitor; ARB, angiotensin II receptor blocker; ARC-HBR, academic research consortium for high bleeding risk; BMI, body mass index; LDL-c, Low-density lipoprotein cholesterol; PCI, percutaneous coronary intervention; PPI, proton pump inhibitor.

**Supplementary Table S2. 1-year clinical endpoint in the intention-to-treat population according to presence of diabetes**

|  | **Diabetes** | | **HR**  **(95%CI)** | ***P***  **value** | **Non-diabetes** | | **HR**  **(95%CI)** | ***P***  **value** | ***P* for**  **interaction** |
| --- | --- | --- | --- | --- | --- | --- | --- | --- | --- |
|  | **Indobufen** | **Aspirin** |  |  | **Indobufen** | **Aspirin** |  |  |  |
| **Primary endpoint^a^** | 40 (4.99) | 53 (6.90) | 0.72 (0.47, 1.08) | 0.110 | 61 (4.19) | 87 (5.70) | 0.73 (0.53, 1.01) | 0.060 | 0.935 |
| **Secondary efficacy endpoint^b^** | 15 (1.87) | 11 (1.43) | 1.31 (0.60, 2.84) | 0.501 | 19 (1.30) | 21 (1.38) | 0.95 (0.51, 1.76) | 0.864 | 0.526 |
| Cardiovascular death | 2 (0.25) | 2 (0.26) | 0.96 (0.13, 6.80) | 0.965 | 1 (0.07) | 2 (0.13) | 0.52 (0.05, 5.78) | 0.598 | 0.703 |
| Nonfatal MI | 3 (0.37) | 4 (0.52) | 0.72 (0.16, 3.21) | 0.665 | 6 (0.41) | 6 (0.39) | 1.05 (0.34, 3.25) | 0.937 | 0.694 |
| Ischemic stroke | 7 (0.87) | 6 (0.78) | 0.72 (0.47, 1.08) | 0.839 | 11 (0.76) | 13 (0.85) | 0.89 (0.40, 1.98) | 0.768 | 0.735 |
| Definite or probable stent thrombosis | 4 (0.50) | 1 (0.13) | 3.82 (0.43, 34.17) | 0.231 | 1 (0.07) | 3 (0.20) | 0.35 (0.04, 3.36) | 0.362 | 0.136 |
| **Secondary safety endpoint^c^** | 25 (3.12) | 42 (5.47) | 0.56 (0.34, 0.92) | 0.023 | 42 (2.88) | 66 (4.33) | 0.66 (0.45, 0.98) | 0.037 | 0.609 |
| BARC type 3, 5 bleeding | 14 (1.75) | 11 (1.43) | 1.20 (0.55, 2.65) | 0.648 | 15 (1.03) | 17 (1.11) | 0.92 (0.46, 1.84) | 0.812 | 0.616 |
| BARC type 2 bleeding | 11 (1.37) | 31 (4.04) | 0.34 (0.17, 0.67) | 0.002 | 27 (1.85) | 49 (3.21) | 0.57 (0.36, 0.92) | 0.021 | 0.206 |
| BARC type 3 bleeding | 13 (1.62) | 10 (1.30) | 1.23 (0.54, 2.80) | 0.626 | 11 (0.76) | 14 (0.92) | 0.82 (0.37, 1.80) | 0.618 | 0.486 |
| BARC type 5 bleeding | 1 (0.12) | 1 (0.13) | 0.95 (0.06, 15.16) | 0.970 | 4 (0.27) | 3 (0.20) | 1.39 (0.31, 6.22) | 0.665 | 0.813 |

Data are expressed as number of patients (percentage).

^a^ The primary endpoint was a composite of cardiovascular death, nonfatal MI, ischemic stroke, definite or probable stent thrombosis, or BARC criteria type 2, 3 or 5 bleeding.

^b^ The secondary efficacy endpoint was a composite of cardiovascular death, nonfatal MI, ischemic stroke, definite or probable stent thrombosis.

^c^ The secondary safety endpoint included BARC criteria type 2, 3 or 5 bleeding.

BARC, Bleeding Academic Research Consortium; CI, confidence interval; HR, hazard ratio; MI, myocardial infarction.

55 Excluded

49 Did not meet eligibility criteria

4 Declined to participate

2 Other Reasons

4606 Assessed for eligibility

4551 Randomized

2258 Randomized to indobufen group

802 with diabetes

1456 without diabetes

2293 Randomized to aspirin group

768 with diabetes

1525 without diabetes

4551 Analyzed in the intention-to-treat population

**Supplementary Figure S1**. Study Flowchart
